# The role of miRNA-32 in non-small cell lung cancer

**DOI:** 10.1097/MD.0000000000049624

**Published:** 2026-07-10

**Authors:** Dongxiao Ding, Haihua Hong, Ke Shi

**Affiliations:** a Department of Thoracic Surgery, The People’s Hospital of Beilun District, Ningbo, Zhejiang, China; b Department of Respiratory Medicine, The People’s Hospital of Beilun District, Ningbo, Zhejiang, China.

**Keywords:** MEF2D, migration, miRNA-32, non-small cell lung cancer, proliferation

## Abstract

**Background:**

This study aimed to investigate whether miRNA-32 affects the proliferation and migration of non-small cell lung cancer (NSCLC) cells by regulating the expression of myocyte enhancer factor 2D (MEF2D).

**Methods:**

Quantitative real-time polymerase chain reaction was utilized to evaluate the expression levels of miRNA-32 in clinical NSCLC tissue specimens and cell lines. Western blotting was employed to detect the protein expression levels of MEF2D, E-cadherin, N-cadherin, and CyclinD1, as well as to verify transfection efficiency. Cell proliferation and migration were assessed using Cell Counting Kit-8 and Transwell assays, respectively. Additionally, a dual-luciferase reporter gene assay was performed to validate the targeted regulatory relationship between miRNA-32 and MEF2D.

**Results:**

miRNA-32 was significantly downregulated in lung cancer tissues and cell lines, whereas MEF2D exhibited significant upregulation. High miRNA-32 expression correlated with poor clinical outcomes in NSCLC across both our in-house cohort and the TCGA cohort. The stable overexpression of miRNA-32 in lung cancer cells markedly inhibited their proliferation and migratory capabilities. Mechanistically, miRNA-32 inhibited the translation of MEF2D by directly binding to its 3’UTR region. Crucially, the overexpression of MEF2D significantly reversed the inhibitory effects of miRNA-32 on lung cancer cell proliferation and migration.

**Conclusion:**

The miRNA-32/MEF2D signaling axis plays a pivotal role in the proliferation and metastasis of NSCLC, highlighting its potential as a diagnostic biomarker and prognostic indicator for the disease.

## 1. Introduction

Lung cancer remains one of the leading causes of cancer-related mortality globally.^[1]^ The incidence is notably higher in industrialized and transitional nations, driven by major risk factors such as smoking, asbestos exposure, and air pollution.^[2]^ The majority of patients present with metastasis at the time of diagnosis, resulting in a 5-year survival rate of <20%.^[3]^ Non-small cell lung cancer (NSCLC) accounts for approximately 80% to 85% of all lung cancer cases.^[4]^ Although targeted therapies and conventional chemotherapy remain standard treatment modalities, overall survival rates have not seen dramatic improvements. The advent of immunotherapy has significantly enhanced patient prognoses and altered the treatment landscape for lung cancer, yielding survival rates up to 5 times higher than those achieved with chemotherapy alone.^[5,6]^ Nevertheless, only a subset of patients derive long-term benefits, with the majority experiencing non-response or disease progression during treatment. Therefore, identifying reliable predictive and prognostic biomarkers remains a critical imperative in the clinical management of lung cancer.

Myocyte enhancer factor 2D (MEF2D), a member of the MEF2 (MADS-box transcription factor) family, functions as a transcription factor regulating growth, development, and signal transduction.^[7]^ While earlier studies primarily linked MEF2D to muscular and nervous system development, recent evidence underscores its involvement in tumorigenesis.^[8,9]^ For instance, MEF2D is highly expressed in gastric cancer, where it correlates with a poor prognosis.^[10]^ Furthermore, Ling et al^[11]^ demonstrated that MEF2D knockdown inhibits β-catenin translocation within the Wnt pathway, thereby suppressing the proliferation and invasion of NSCLC cells. Multiple studies have also indicated that MEF2D is targeted and regulated by various miRNAs during lung cancer progression.^[12–14]^ Collectively, these findings suggest that MEF2D plays a critical oncogenic role in NSCLC.

miRNAs are a class of small (about 22 nucleotides) non-coding RNAs that participate in the regulation of gene expression and cell communication at multiple levels.^[15,16]^ miRNAs exert their regulatory function by binding to complementary or near-complementary sequences in the 3’ untranslated region (3’UTR) of target mRNAs, which may lead to mRNA degradation or decreased protein translation efficiency.^[17,18]^ Recent studies have shown that the ectopic expression of miRNAs is usually related to the occurrence and development of tumors, such as participating in cancer cell proliferation, migration, invasion, apoptosis, and the cell cycle.^[19–22]^ miRNA-32 has been shown to be involved in the development of various cancers, such as glioma, prostate cancer, and multiple myeloma.^[21,23,24]^ In addition, there are relevant reports on the mechanism of miRNA-32 in lung cancer.^[25–29]^ However, whether miRNA-32 can affect the proliferation and migration abilities of lung cancer cells by targeting the expression level of MEF2D has not been reported.

Therefore, this study aimed to explore the mechanism by which miRNA-32 inhibited the proliferation and migration of lung cancer cells, and further investigate whether miRNA-32 and MEF2D have a regulatory role in the proliferation and migration of lung adenocarcinoma (LUAD), in order to provide an important theoretical basis for the clinical treatment of NSCLC and the development of targeted drugs.

## 2. Materials and methods

### 2.1. Clinical samples

Tumor tissue samples and adjacent non-cancerous tissue samples were collected from 20 patients with NSCLC from Ningbo Beilun District People’s Hospital (Beilun Branch, No. 1 Hospital Affiliated to Medical College of Zhejiang University). This study was approved by the Medical Ethics Committee of Ningbo Beilun District People’s Hospital. The pathological biopsy of the tissue before and after the operation was confirmed as NSCLC. The collected tissue was fixed with 4% paraformaldehyde, dehydrated with alcohol gradient, and prepared into tissue wax blocks for subsequent experiments.

### 2.2. Plasmids

miRNA-32 and MEF2D overexpression plasmids were purchased from Tsingke Biotechnology Co., Ltd. (China), and the control plasmid PCDH was purchased from Guangzhou Geneseed Biotech Co., Ltd. (China).

### 2.3. Cell culture

Human normal bronchial epithelial cells BEAS-2B were purchased from BeNa Culture Collection (BNCC359274, China), human kidney epithelial cells 293T (CRL-3216), NSCLC cells A549 (CRM-CCL-185), and NCI-H1299 (CRL-5803) were purchased from ATCC (USA). Cells were cultured in RPM-I640 medium (Sigma, USA) containing 10% fetal bovine serum and placed in a 37°C, 5% CO_2_ incubator.

### 2.4. Cell transfection

miRNA-32 mimic was purchased from Genechem (China), oe-MEF2D was purchased from Tsingke Biotechnology Co., Ltd. (China), and the negative control plasmid was purchased from Guangzhou Geneseed Biotech Co., Ltd. (China). Cells were seeded at a density of 3 × 10^5^ cells/well in a 6-well plate, and transfected with Lipofectamine 2000 (Invitrogen, Carlsbad, CA) according to the manufacturer’s instructions. Cells were collected 48 hours after transfection for subsequent experiments.

### 2.5. Quantitative real-time polymerase chain reaction (qRT-PCR)

Total RNA was extracted from the cultured cells using TRIzol reagent (Ambition, USA). Complementary DNA (cDNA) was synthesized via reverse transcription using the GoScript™ Reverse Transcription System (A5001, Promega, USA) according to the manufacturer’s guidelines. PCR amplification was performed utilizing the GoTaq® qPCR Master Mix (Promega, USA). The qPCR cycling parameters were defined as follows: initial activation at 95°C for 2 minutes; followed by 40 cycles of denaturation at 95°C for 15 seconds, annealing at 60°C for 30 seconds, and extension at 60°C for 30 seconds. The reactions were executed using the StepOne™ Real-Time PCR System (Applied Biosystems, USA). U6 and GAPDH were utilized as the internal controls for miRNA and mRNA normalization, respectively, and the relative expression levels were calculated employing the 2^−ΔΔCt^ method. The primer sequences used in this study are detailed in Table 1.

**Table 1:** qRT-PCR primers.

Genes	Primer	
miR-32	Forward primer	5’- CGGTATTGCACATTACTAAGTTGCA -3’
	Reverse primer	5’- CTCGCTTCGGCAGCACA -3’
MEF2D	Forward primer	5’- CGTGCTATGTGACTGCGAGAT-3’
	Reverse Primer	5’- GCGTCGGTACTTGTCCTCC -3’
U6	Forward primer	5′-GCTTCGGCAGCACATATACTAAA-3′
	Reverse primer	5′-CGCTTCACGAATTTGCCGTG-3′
GAPDH	Forward primer	5’- ATCAATGGAAATCCCATCACCA -3’
	Reverse primer	5’- GACTCCACGACGTACTCAGCG -3’

### 2.6. potential target of miRNA-32

Potential targets of miRNA-32 were predicted by several miRNA-based databases, including miRDB, Targetminer, miRTarBase and miRWalk.

### 2.7. Western blot

Total proteins were extracted from cells using RIPA lysis buffer (Beyotime, China), and the protein concentration of the extracted samples was measured using the BCA protein concentration assay kit (Beyotime, China). Protein samples were separated using 10% SDS-polyacrylamide gel electrophoresis, and then transferred onto polyvinylidene difluoride membranes (Millipore, USA). The membranes were blocked with 5% skim milk for 1 hour, washed with Tris-Buffered Saline with Tween-20, and then incubated overnight at 4°C with primary antibodies, including rabbit anti-E-cadherin (weight: 120 KDa, Cell Signaling Technology, USA), N-cadherin (weight: 130 KDa, Abcam, USA), CyclinD1 (weight: 34 KDa, Cell Signaling Technology, USA), MEF2D (weight: 56KDa, Cell Signaling Technology, USA), CYYR1 (weight: 35 KDa, Cell Signaling Technology, USA), and GAPDH (weight: 36 KDa, Proteintech, USA).

The membranes were then washed with tris‑buffered saline with Tween‑20 and incubated with secondary IgG antibodies (Abcam, UK). The protein bands of interest were visualized using an ECL assay kit (Beyotime, China) and the Typhoon FLA7000 imager, and analyzed using a gel image processing system.

### 2.8. Dual-luciferase reporter assay

The 3’UTR fragment of MEF2D containing the predicted miRNA-32 binding site (wild-type, WT) or a mutated binding site (mutant, MUT) was synthesized and cloned into the pmirGLO dual-luciferase reporter vector (Promega, USA). For the luciferase assay, 293T cells were seeded in 24-well plates at a density of 5 × 10^4^ cells/well and cultured overnight. Cells were co-transfected with 100 ng of the WT or MUT reporter plasmid, 50 nM of miRNA-32 mimic or mimic-NC, and 10 ng of pRL-TK Renilla luciferase control vector (Promega, USA) using Lipofectamine 2000 (Invitrogen, USA). After 48 hours of transfection, cells were lysed using 1 × Passive Lysis Buffer (Promega, USA), and firefly and Renilla luciferase activities were measured sequentially using the Dual-Luciferase® Reporter Assay System (Promega, USA) on a GloMax® 20/20 Luminometer (Promega, USA). Firefly luciferase activity was normalized to Renilla luciferase activity for each well. Each experiment was performed in triplicate with 3 independent replicates. The relative luciferase activity was calculated as the ratio of normalized firefly luminescence in the miRNA-32 mimic group to that in the mimic-NC group.

### 2.9. Cell counting kit-8 (CCK-8) assay

Cell proliferation was assessed by cell counting kit-8 (CCK-8) (US Everbright Inc.) assays using an ELISA microplate reader (Bio-Rad Laboratories, Inc., Hercules, CA). LUAD cells were seeded in a 96-well plate at a density of 1 × 10^4^ cells/well and cultured at 37°C with 5% CO_2_ and incubated for 0, 24, 48 or 72 hours. According to the manufacturer’s protocol, the cells of each well were added with 10 μL CCK-8 (US Everbright Inc.) and cultured at 37°C for 2 hours. The absorbance of each well was measured at 450 nm using a microplate reader, and the relative cell viability was calculated.

### 2.10. Transwell assay

Cells (5 × 10^4^) were seeded in the upper chamber (8 μm, Corning, USA) of a serum-free culture medium without Matrigel. The lower chamber was supplied with medium containing 10% fetal bovine serum. After 24 hours of incubation, cells on the upper surface of the chamber were gently wiped with a cotton swab, fixed with 4% paraformaldehyde, and stained with 0.1% crystal violet. Finally, the cells in the lower chamber were counted by randomly selecting 3 to 5 microscopic fields.

### 2.11. Statistical analysis

All experimental data were expressed as mean ± standard deviation (χ̄ ± SD) or SEM (n ≥ 3), and data processing was performed using SPSS 18.0 statistical software and GraphPad Prism 7.0 software for plotting. Student *t* test was used to analyze differences between 2 groups, and one-way analysis of variance followed by Tukey’s post hoc test was used to analyze differences among multiple groups. *P < .05 was considered statistically significant.

## 3. Results

### 3.1. The expression and prognostic value of miRNA-32 and MEF2D in NSCLC

**Figure 1.**
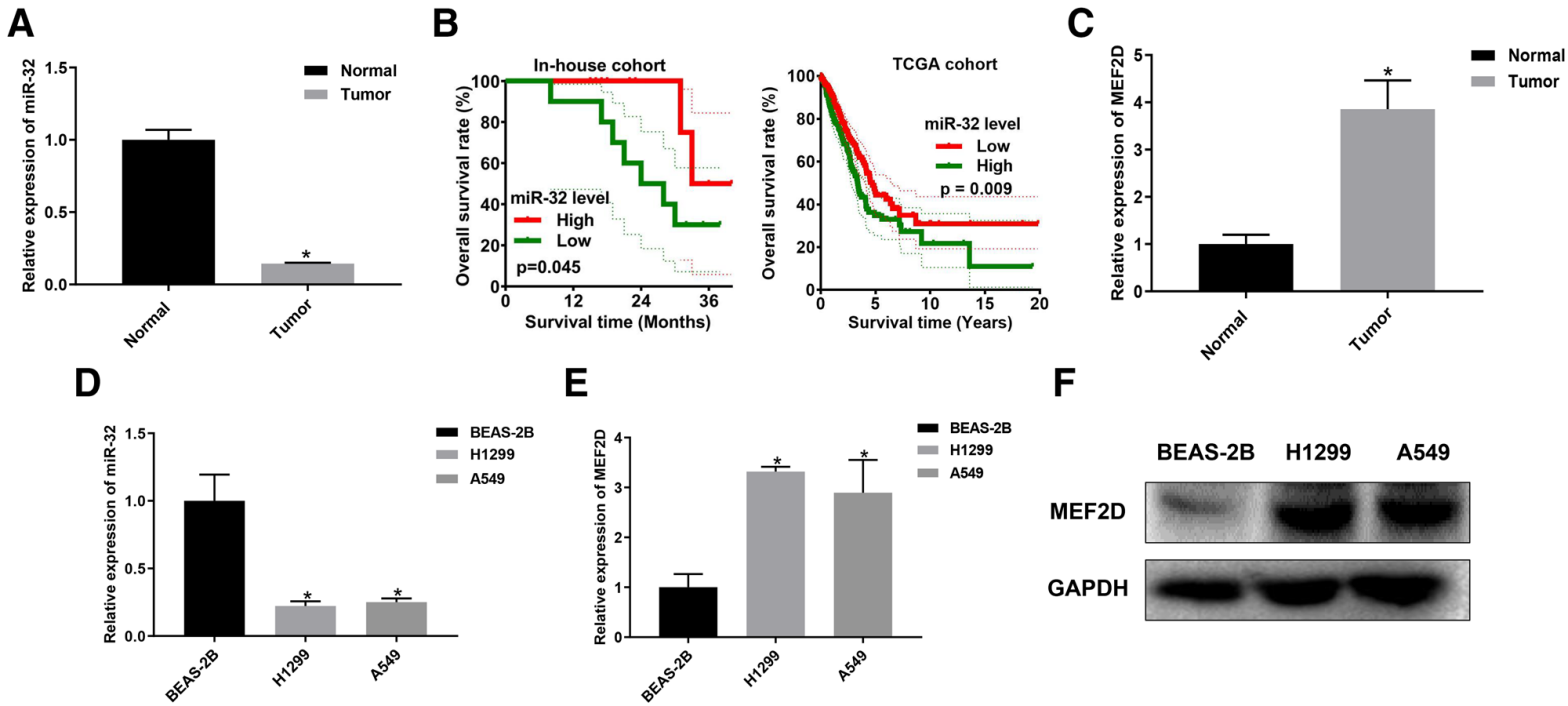
Differential expression of miRNA-32 and MEF2D in human lung cancer tissues. (A-B) The expression and prognostic value of miRNA-32 in lung cancer. (C) MEF2D mRNA expression levels were detected by qRT-PCR in lung cancer tissues. (D) miRNA-32 expression and (E) MEF2D mRNA expression levels were detected by qRT-PCR in human lung cancer cell lines. (F) Protein expression levels of MEF2D in human lung cancer cell lines were detected by Western blot. * Indicates significant difference compared to the control group (*P* < .05).

miRNA microarray studies have shown that miRNA-32 is downregulated in LUAD.^[30]^ In addition, research has shown that MEF2D is highly expressed in chronic obstructive pulmonary disease patients with NSCLC and may be a potential biomarker during chronic validation and lung cancer transition.^[31]^ Therefore, to further verify the expression levels of miRNA-32 and MEF2D in NSCLC, we collected 20 lung cancer tissues and their adjacent tissues and detected the expression levels of miRNA-32 and MEF2D by qRT-PCR. Table 2 shows the characteristics of 20 lung cancer cases. The results showed that compared with adjacent tissues, miRNA-32 was significantly downregulated in lung cancer tissues (*P* < .05, Figure 1A). We also found that high miRNA-32 expression was correlated with poor clinical outcome in NSCLC in both in-house and TCGA cohorts (Fig. 1B). MEF2D was significantly upregulated in lung cancer tissues (Fig. 1C). Furthermore, the expression levels of miRNA-32 and MEF2D showed the same results in lung cancer cell lines (Figs. 1D-F). This result suggested that miRNA-32 and MEF2D may play an important role in the progression of lung cancer.

**Table 2:** Participants characteristics.

	Healthy control (N = 20)
Age (yr)	65.23 ± 8.54
Gender	
Male	11
Female	9
Clinical stage	
I	5
II	9
III	6
Grade	
Low	12
High	8
Lymph node metastasis	
Positive	14
Negative	6

### 3.2. Overexpression of miRNA-32 significantly inhibits the proliferation and migration abilities of lung cancer cells

**Figure 2.**
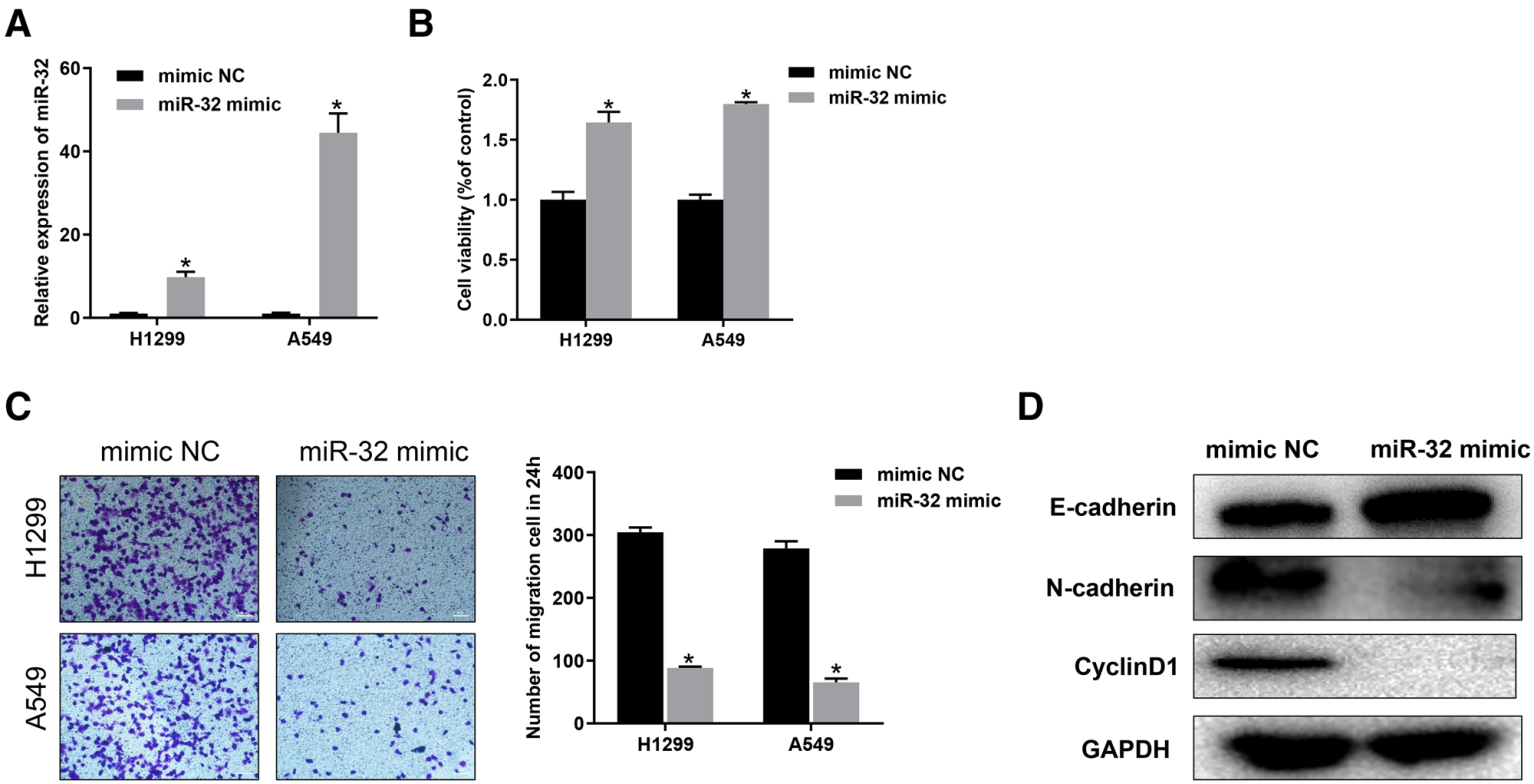
Overexpression of miRNA-32 significantly inhibits the proliferation ability of lung cancer cells. (A) The transfection efficiency of miRNA-32 in lung cancer cells was detected by qRT-PCR. (B) CCK-8 assay was performed to detect the effect of miRNA-32 overexpression on the proliferation ability of H1299 and A549 cells. (C) A Transwell assay was performed to detect the effect of miRNA-32 overexpression on the migration ability of H1299 and A549 cells. (D) The effect of miRNA-32 overexpression on the expression of N-cadherin, E-cadherin, and CyclinD1 proteins was detected by Western blot. *Indicates significant difference compared to the control group (*P* < .05).

To further investigate the function of miRNA-32 in lung cancer cells, we first transfected miRNA-32 mimic and mimic NC plasmids into H1299 and A549 cells and detected the transfection efficiency by qRT-PCR. The results showed that miRNA-32 was significantly upregulated in both H1299 and A549 cells (Fig. 2A). Subsequently, we detected cell viability by CCK-8 assay. The results showed that overexpression of miRNA-32 significantly inhibited the proliferation ability of H1299 and A549 cells (Fig. 2B). Next, we used the Transwell assay to detect the effect of miRNA-32 overexpression on cell migration ability. The results, as shown in Figure 2C, showed that compared with the control group, overexpression of miRNA-32 significantly inhibited the migration ability of lung cancer cells. In addition, we also detected the expression of the tumor cell proliferation-related protein CyclinD1 and the metastasis-related proteins N-cadherin and E-cadherin. The results showed that overexpression of miRNA-32 inhibited the expression of N-cadherin and CyclinD1, but promoted the expression of E-cadherin (Fig. 2D).

### 3.3. miRNA-32 inhibits the translation of MEF2D by targeting its 3’UTR region and thereby inhibits its expression

**Figure 3.**
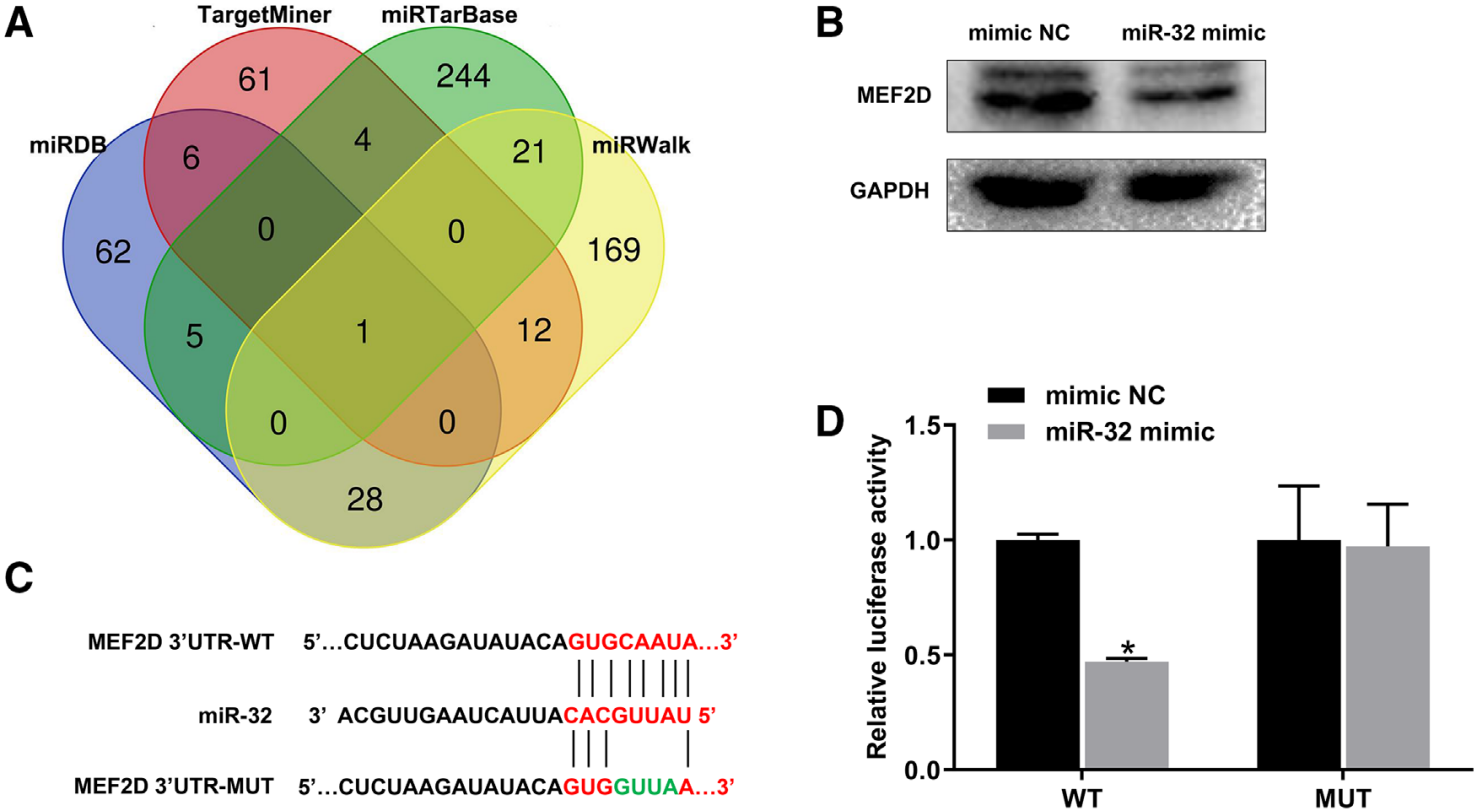
miRNA-32 inhibits MEF2D expression by binding to its 3’UTR region. (A) The targets of miRNA-32 predicted by miRDB, Targetminer, miRTarBase and miRWalk. (B) Protein expression levels of MEF2D were detected by Western blot. (C) Alignment of miRNA-32 and MEF2D seed sequences. (D) Dual-luciferase reporter gene assay was performed to detect the luciferase intensity. * Indicates significant difference compared to the control group (*P* < .05).

As shown in Figure 3A, MEF2D were suggested as the target of miRNA-32. We have demonstrated that miRNA-32 has a significant inhibitory function in the proliferation and migration of lung cancer cells, and that the expression of miRNA-32 and MEF2D in lung cancer tissues and cell lines showed opposite trends. It is well known that one of the important functions of miRNA is to inhibit the translation of target gene mRNA by binding to the 3’UTR region of the target gene mRNA using its seed sequence, thereby ultimately inhibiting the protein expression of the target gene. Therefore, we speculated that miRNA-32 may affect the expression of MEF2D in the same way. First, we found through western blot that the expression of MEF2D in the miRNA-32 mimic group was significantly reduced compared to the mimic NC group (Fig. 3B), which is consistent with our speculation. To further confirm the relationship between miRNA-32 and MEF2D, we first predicted the potential binding sites of miRNA-32 and MEF2D 3’UTR regions (Fig. 3C). The dual-luciferase reporter assay showed that co-transfection with miRNA-32 mimic significantly reduced the relative luciferase activity of the wild-type reporter to 42.3% ± 5.1% of the mimic-NC control group (*P* < .01). In contrast, mutation of the predicted binding site completely abolished this effect, with the mutant reporter showing no significant difference in luciferase activity between the miRNA-32 mimic and mimic-NC groups (96.7% ± 6.2% of control, *P* > .05, Figure 3D). In summary, miRNA-32 could target and regulate the expression of MEF2D.

### 3.4. miRNA-32 promotes the proliferation and migration abilities of lung cancer cells by targeting and regulating MEF2D

**Figure 4.**
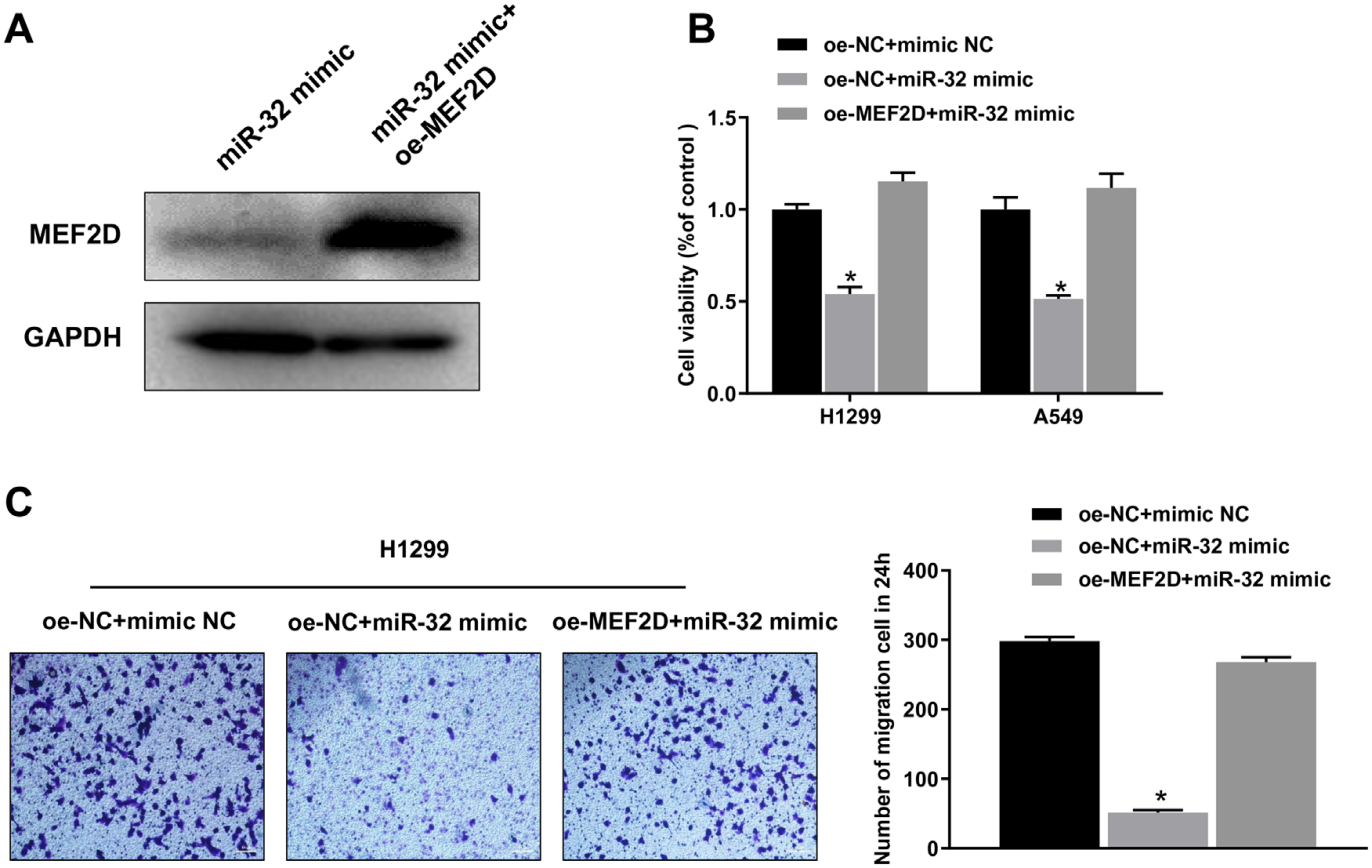
MEF2D promotes the proliferation and migration abilities of lung cancer cells. (A) Expression levels of MEF2D in cell lysates were detected by Western blot. (B) CCK-8 assay was performed to detect the effect of MEF2D on the proliferation ability of H1299 and A549 cells. (C) A Transwell assay was performed to detect the effect of MEF2D on the migration ability of H1299 and A549 cells. * Indicates significant difference compared to the control group (*P* < .05).

To further explore whether miRNA-32 can affect the proliferation and migration abilities of lung cancer cells by targeting and regulating the expression of MEF2D, we first overexpressed miRNA-32 and MEF2D simultaneously in H1299 cells and detected the overexpression efficiency by Western blot. The results showed that overexpression of MEF2D could restore the inhibitory effect of miRNA-32 overexpression on MEF2D expression (Fig. 4A). Subsequently, we detected cell viability by CCK-8 assay and found that the inhibitory effect of miRNA-32 on cell proliferation was partially rescued by MEF2D overexpression (Fig. 4B). Moreover, Transwell assay showed that overexpression of MEF2D could also partially rescue the inhibitory effect of miRNA-32 on cell migration (Fig. 4C). These results suggested that miRNA-32 could promote the proliferation and migration abilities of lung cancer cells by targeting and regulating MEF2D.

## 4. Discussion

Despite significant progress in research on tumor treatment, lung cancer remains one of the most common causes of cancer-related death globally.^[32]^ In recent years, multiple miRNAs have been identified as key regulatory factors in the occurrence and development of lung cancer, with therapeutic potential.^[33–35]^ However, the molecular mechanism of miRNAs in NSCLC is not fully understood.

miRNA-32 is an intronic microRNA expressed in various tissues in humans and mice, including serum, liver, kidney, breast, and brain tissues.^[36–39]^ Wu et al^[40]^ revealed the structure and regulation of the hsa-miRNA-32 promoter using multiple molecular biology methods and found that transcription factors SMAD1, STAT1, and Foxk1 may be involved in the transcriptional regulation of miRNA-32. Furthermore, studies have found that miRNA-32 has been confirmed to play a role in the progression of tumors. Li et al^[25]^ found that miRNA-32 is downregulated in NSCLC and promotes the proliferation, migration, and epithelial-mesenchymal transition process of NSCLC cells. In addition, the study also showed that miRNA-32 directly targets TWIST1 to inhibit the malignancy of NSCLC. Zheng et al^[29]^ found that miRNA-32 is downregulated in diamminedichloroplatinum (DDP)-resistant NSCLC and inhibits DDP sensitivity by targeting ROBO1. Furthermore, miRNA-32/ROBO1 promotes the sensitivity of NSCLC cells to DDP by inhibiting the Wnt/β-catenin signaling pathway. In this study, we also found that miRNA-32 was significantly downregulated in lung cancer tissues and lung cancer cells compared to adjacent tissues. Subsequently, overexpression of miRNA-32 was found to inhibit the proliferation and migration abilities of H1299 and A549 cells, indicating that miRNA-32 played an important role in the malignant progression of LUAD.

Studies have reported that the signal expression of MEF2D is elevated in several human cancers, and upregulation of MEF2D is believed to contribute to cancer development. For example, researchers found that MEF2D expression is increased in pancreatic cancer tissues, and the increase in MEF2D expression is related to the tumor size, histological differentiation, and TNM stage of pancreatic cancer patients.^[41]^ In addition, MEF2D expression is an independent prognostic indicator for pancreatic cancer patients. Nianxu Luan *et al*^[14]^ found that MEF2D is abnormally expressed in lung cancer, and the expression of MEF2D and miR-30a in lung cancer is negatively correlated. Meanwhile, their experimental results showed that miR-30a inhibits lung cancer cell proliferation and induces tumor cell apoptosis by directly targeting the 3’UTR of MEF2D mRNA. In this study, we found that overexpression of MEF2D promoted the proliferation and migration abilities of lung cancer cells. Combined with the abnormal expression of miRNA-32 and MEF2D in lung cancer tissues and cells, further analysis revealed a negative correlation trend between their expressions, and overexpression of miRNA-32 significantly reduced MEF2D expression. Further experimental studies found that miRNA-32 and the 3’UTR region of MEF2D directly interacted with each other, thus further confirming that MEF2D was a direct binding molecule of miRNA-32. Cell functional experiments showed that miRNA-32 targeted regulation of MEF2D expression to inhibit the proliferation and migration abilities of lung cancer cells.

Although the study found that miRNA-32 could inhibit the metastasis of NSCLC and suppress the proliferation and migration of lung cancer cells by inhibiting MEF2D expression, there are still some shortcomings in this study: The regulatory mechanism of miRNA is diverse, and whether miRNA-32 has other pathways, such as direct binding with proteins, affecting cell proliferation and migration, is unknown. Due to the short sample collection time and incomplete follow-up information, the survival curve of miRNA-32 was not drawn, which could have provided important evidence to support whether miRNA-32 can be used as a good prognostic marker. This study found that miRNA-32 was of great significance in the proliferation and migration of NSCLC, and whether it could be detected in serum or exosomes needs to be explored in future experiments.

In summary, this study further deepens our understanding of miRNA-32 and MEF2D, with the aim of providing important theoretical basis for the clinical treatment of NSCLC and the development of targeted drugs.

## 5. Conclusion

The miRNA-32/MEF2D signaling axis plays an important role in the proliferation and migration of NSCLC and is expected to become a biomarker for diagnosing and predicting the prognosis of NSCLC.

## Author contributions

**Investigation:** Dongxiao Ding.

**Formal analysis:** Haihua Hong.

**Project administration:** Ke Shi.

**Writing – original draft:** Dongxiao Ding.

**Writing – review & editing:** Ke Shi.
